# A TECHNOLOGY-BASED BODY-MIND INTERVENTION FOR LOW-INCOME AMERICAN OLDER ADULTS

**DOI:** 10.1093/geroni/igac059.1080

**Published:** 2022-12-20

**Authors:** Ladda Thiamwong, Rui Xie, Joon-Hyuk Park, Nichole Lighthall, Victoria Loerzel, Jeffrey Stout

**Affiliations:** University of Central Florida, Orlando, Florida, United States; University of Central Florida, Orlando, Florida, United States; University of Central Florida, Orlando, Florida, United States; University of Central Florida, Orlando, Florida, United States; College of Nursing, University of Central Florida, Orlando, Florida, United States; University of Central Florida, Orlando, Florida, United States

## Abstract

Research is limited on the use of technology to help individuals who have a mismatch between physiological fall risk (Body) and perceived fall risk (Mind) and are unable to access traditional fall interventions. We examined the feasibility and acceptability of a technology-based body-mind intervention in low-income older adults during the COVID-19 pandemic and explored barriers to access and adopting the technology. Data were collected using a survey, balance test, accelerometer-based physical activity (PA), and semi-structured interviews with twenty participants who engaged in an 8-week intervention at a low-income setting in Florida. We found that: 1) the technology-based intervention is feasible, 2) participants tend to accept technology to alter their perceptions of fall risk and balance capacity, 3) tailored activities to each component are not a one-size-fits-all approach. There were no statistically significant changes in sedentary time, light PA, and moderate to vigorous PA between pre and post-intervention.

